# Scalable ω‐Substituted α‐Olefins via Living Chain Transfer Telomerization: A Platform for Next Generation Polyolefins

**DOI:** 10.1002/anie.6963058

**Published:** 2026-08-07

**Authors:** Lauren G. Logue, Cole M. Burrows, Vince J. Wedekind, Hyunku C. Kim, Lawrence R. Sita

**Affiliations:** ^1^ Department of Chemistry and Biochemistry University of Maryland MD USA

**Keywords:** functional, iving polymerization, polyolefinl

## Abstract

Isomerically pure, ω‐substituted α‐olefins of general structure, X‐(CH_2_)_m_(CH_2_CH_2_)*_n_*_‐1_CH = CH_2_ (*m* = 0 or 1; X = alkyl or aryl) (**3**), have been produced in practical and scalable quantities as narrow Poisson molar mass distributions of separable *n*‐mers (e.g. *n* = 1 – 10) through a one‐pot, two‐step process involving: living ternary chain transfer telomerization of tris(ω‐substituted alkyl)aluminum, [X‐(CH_2_)_m_CH_2_CH_2_]_3_Al (**1**), with ethene and a small amount of diethylzinc as a chain transfer mediator, using an in situ generated ion pair initiator derived from the cyclopentadienyl, amidinate (CPAM) Hf pre‐initiator, (η^5^‐C_5_Me_5_)[κ^2^‐(*N,N’*)‐N(Et)C(Me)N(Et)]HfMe_2_ (**2**) and the borate co‐initiator, [PhNHMe_2_][B(C_6_F_5_)_4_] (**B**), followed by catalytic displacement of the trialkylaluminum intermediate using bis(1,5‐cyclooctadiene)Ni(0) as the pre‐catalyst. Results are further presented for production of several families of “next generation” polyolefins that are obtained through CPAM Zr‐ and Hf‐mediated stereoselective (enantioselective) living coordination polymerization of different derivatives of **3** and including copolymerization with a variety of linear and branched α‐olefins and 1,5‐hexadiene.

## Graphical abstract

A highly versatile and general strategy is presented for the scalable production of practical quantities of ω‐substituted α‐olefins using tris(ω‐substituted alkyl)aluminum telogens that are readily available through a “green” synthesis from commodity triisobutylaluminum and ethene as the taxogen. These new α‐olefins can be designed and used as monomers for next generation polyolefins that have increased range of functionality and tunable physical properties.

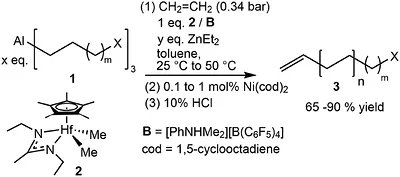

## 1 Introduction

Polyolefins represent the largest class of synthetic materials ever produced, with global production exceeding 200 million metric tons annually [1, 2, 3, 4]. Despite this enormous scale and technological importance, the structural diversity of polyolefins remains surprisingly limited. More to the point, most commercial polyolefin materials are derived from only a small set of α‐olefin monomers—principally ethene, propene, and a few linear α‐olefin (LAO) comonomers, such as 1‐hexene and 1‐octene [5]. Indeed, while many synthetically intriguing α‐olefins can be prepared on the laboratory scale, very few synthetic routes exist that can produce new classes of monomers in quantities commensurate with the industrial scale at which polyolefins are produced. Additionally, these monomers must have critically high isomeric purity for terminal unsaturation as insertion of internal alkenes often results in chain termination. As a result, the design space for next generation polyolefin materials has remained tightly coupled with the development and availability of new scalable olefin monomer platforms [6, 7, 8].

One historical advance illustrates the importance of scalable monomer synthesis. In 1960, Karl Ziegler and coworkers [9] introduced the *Aufbaureaktion* (“building‐up reaction”) for the industrial production of isomerically pure linear α‐olefins (LAOs) (**I**). As summarized in Scheme 1, this process proceeds through controlled telomerization [10] of triethylaluminum (AlEt_3_) serving as the telogen and ethene as the taxogen under high pressure and moderate temperature (e.g. 120 bar, 100°C) to produce the intermediate chain‐grown trialkylaluminum intermediates **II**. An increase in temperature and reduction in ethene pressure then effects chain displacement to provide **I** with regeneration of AlEt_3_. Importantly, the initially produced narrow Poisson distribution of *n*‐mers of **I** can either be used “as is” or separated into pure *n*‐mer components through fractional distillation. LAOs are currently manufactured on the billions of pounds per year scale and have become critical feedstocks across the chemical industry, which includes providing α‐olefin comonomers for polyolefins [11, 12, 13]. Remarkably, despite more than seventy years of development in organometallic catalysis and polymer chemistry, no analogous scalable method has ever emerged for the controlled telomerization of *substituted* trialkylaluminum telogens that could provide practical access to new classes of precisely substituted 1‐alkenes. Consequently, the molecular diversity of α‐olefin monomers available for polyolefin synthesis has remained severely restricted to LAOs and 4‐methyl‐1‐pentene (4M1P), which is obtained through the dimerization of propene [14, 15]. Herein, we now report that this longstanding gap can be addressed through the living ternary coordinative chain transfer telomerization (LTCCT) process of Scheme 2 in which tris(ω‐substituted alkyl)aluminum telogens (**1**) undergo formal ethene insertions into all three Al‐C bonds, as mediated by the ion pair initiator derived from “activation” of the cyclopentadienyl, amidinate (CPAM) pre‐initiator, (η^5^‐C_5_Me_5_)[κ^2^‐(*N,N’*)‐N(Et)C(Me)N(Et)]HfMe_2_ (**2**), by the borate co‐initiator. [PhNHMe_2_][B(C_6_F_5_)_4_] (**B**). A reactive quench involving Ni‐catalyzed displacement under an ethene atmosphere then provides excellent yields of the corresponding isomerically pure ω‐substituted α‐olefins (**3**) as a narrow Poisson distribution of separable *n*‐mers [16]. We further demonstrate that this new class of ω‐substituted α‐olefins are viable monomers and comonomers for production of “next generation” polyolefins via living coordination polymerization (LCP). Finally, the tris(ω‐substituted alkyl)aluminum telogens (**1**) are obtained through a simple one‐step solventless, no‐chemical‐waste‐generated “green” process involving thermal transalkylation of industrially available triisobutylaluminum (TIBAL), Al(iBu)_3_, and 1‐alkenes according to Scheme 3 [17]. The scalability of this process and LTCCT helps to ensure future accessibility of practical quantities of a wide range of derivatives of **3** and next generation polyolefins derived therefrom.

**SCHEME 1:**
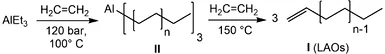
Industrial process for production of linear α‐olefins (LAOs) (**I**).

**SCHEME 2:**
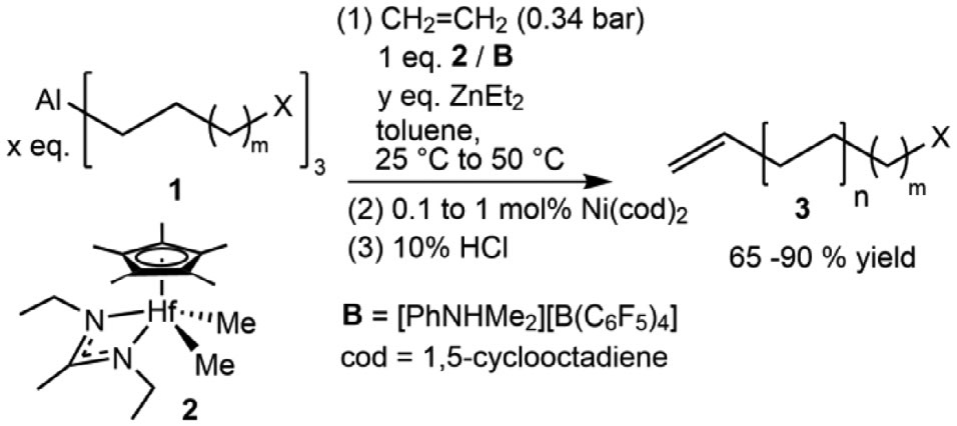
Transition‐metal‐mediated living ternary chain transfer telomerization of tris(ω‐substituted alkyl)aluminum (**1**) by ethene followed by Ni‐catalyzed displacement to produce isomerically pure ω‐substituted α‐olefins (**3**).

**SCHEME 3:**
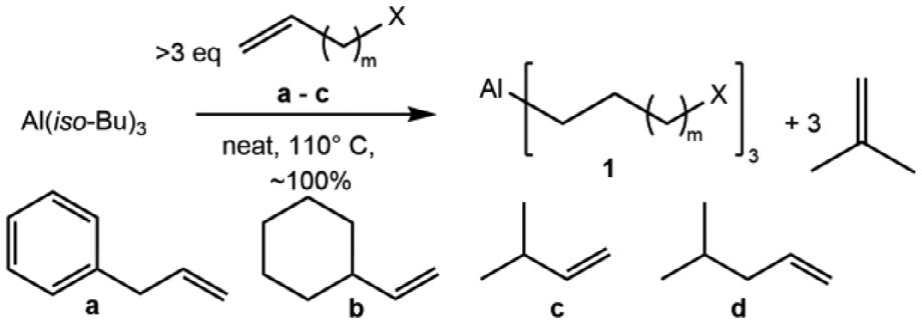
Solventless transalkylation of TIBAL with representative 1‐alkenes to provide the tris(ω‐substituted alkyl)aluminum telogens (**1**).

## 2 Results and Discussion

Recently we introduced LTCCT as a means by which to produce isomerically pure ω‐substituted fatty alcohols (**III**) and acids (**IV**) from the same starting tris(ω‐substituted alkyl)aluminum telogens (**1**) according to the top part of Scheme 4 [17]. LTCCT represents the latest evolution of our well‐established methods for LCP and living coordinative chain transfer polymerization (LCCTP) mediated by CPAM group 4 metal initiators that have been published over the past 25 years [18, 19, 20, 21, 22, 23, 24, 25]. The bottom half of Scheme 4 presents the key mechanistic steps involved in LTCTT, which starts with in situ activation of **2** through methyl group protonolysis by the anilinium cation of borate **B** to generate the active ion pair initiator, {(η^5^‐C_5_Me_5_)[κ^2^‐(*N,N’*)‐N(Et)C(Me)N(Et)]HfMe}] [B(C_6_F_5_)_4_] (**V**) (see equation 1 in Scheme 4b). In the absence of a chain transfer agent (CTA), LCP proceeds with an α‐olefin through standard chain growth propagation involving monomer complexation and migratory insertion. A reactive quench with acid provides a H‐terminated polyolefin with tuneable number‐average degree of polymerization and molar mass index, *DP_n_* and *M_n_*, respectively, and a very narrow molar mass distribution as quantified by a typically small dispersity index, *Ð*, of ≤ 1.1 [26]. We developed LCCTP as a workaround solution to the formal one‐chain‐per‐active‐site limitation of LCP [22, 23, 24, 25]. More specifically, addition of a stoichiometric excess of a main‐group‐metal alkyl, such as diethylzinc (ZnEt_2_), as a CTA provides a large population of “surrogate” chain growth centers that engage in rapid and reversible chain exchange with the much smaller population of active transition‐metal propagator. Under the conditions where the rate and rate constant for chain exchange, ν_CT_ and *k*_CT_, are far greater in magnitude than those for chain propagation, ν_p_ and *k*_p_, then all the desired features of a living polymerization can be achieved [22, 23, 24, 25, 26]. However, the scalability of the process is now dependent only on the cost and availability of the main‐group‐metal alkyl, which in the case of ZnEt_2_ is routinely shipped in rail car quantities. To capitalize further on AlEt_3_ as a cheaper, more abundant, and less pyrophoric CTA, we further developed living *ternary* chain transfer polymerization that makes use of the relative rates of chain transfer between metal species according to the order: Zn ↔ Al > Hf ↔ Zn >> Hf ↔ Al (see equation 2 in Scheme 4b). Thus, while AlEt_3_ by itself is not an effective CTA due to very slow Hf ↔ Al chain exchange that causes a significant increase in dispersity, addition of a small amount of ZnEt_2_ can mediate rapid and reversible chain exchange between Hf and Al by virtue of very fast Zn ↔ Al and Hf ↔ Zn reversible chain exchange [24]. In this mechanism, a small amount of ZnEt_2_ relative to AlR’_3_ and initiator V serves as a ‘chain transfer mediator’. In practice, a AlR’_3_ / ZnEt_2_ ratio of 19:1 ensures that the level of Et groups introduced by ZnEt_2_ is only about 3% of the total. Further, we have demonstrated that each alkyl group that is initially present on the Hf, Al, and Zn reagents contributes to the total number of exchanging and growing polymer chains, which can be used to target a specific *DP*_n_ and *M*_n_ of a final product by setting the amount of monomer consumed (see equation 3 in Scheme 4b).

**SCHEME 4:**
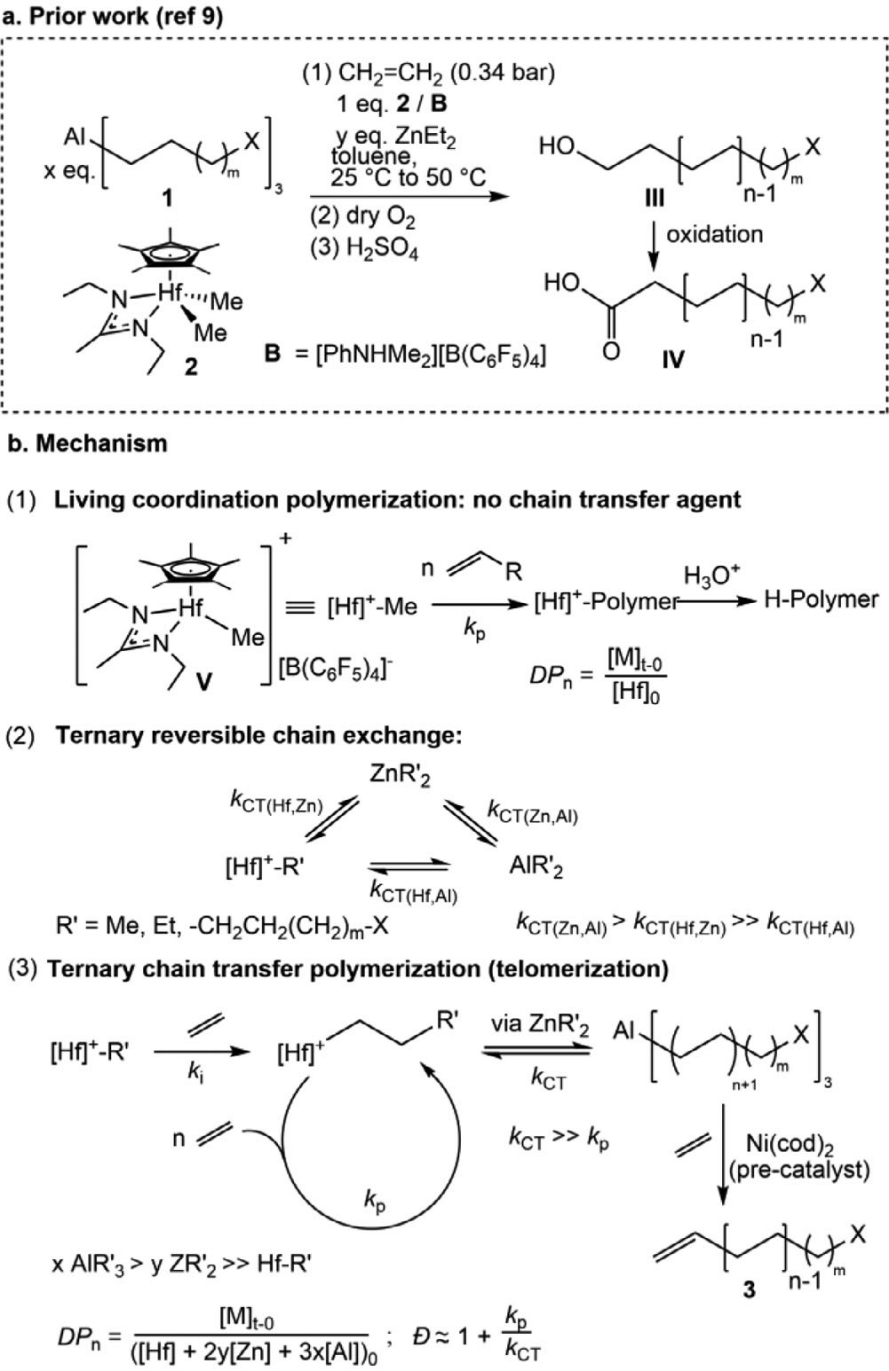
(top) living ternary chain transfer telomerization of tris(ω‐substituted alkyl)aluminum (**1**) to produce ω‐substituted fatty alcohols (**III**) and fatty acids (**IV**) and (bottom) key mechanistic steps involved in LCP, LCCTP and ternary chain exchange.

The initiating chain ends of the polyolefin will be a mix of Me and Et groups of **V** and ZnEt_2_ employed as initiator and CTM, respectively, and the R’ group provided by the AlR’_3_ telogen **1**. In practice, a ratio of 190: 10: 1 for **1**: ZnEt_2_: **2** ensures that 97% of chains will be capped by R’. This targeted structural (isomeric) purity of product can be increased further by reducing the amount of ZnEt_2_ employed and the present work does not attempt to provide the optimum ratio. Finally, we have previously reported that low to very low molar mass end‐group‐functionalized polyolefins with *DP*_n_ values of 5 ‐ 15 can be obtained at high CTA / initiator ratios with a variety of α‐olefin and α,ω‐non‐conjugated dienes [27, 28]. The living ternary telomerization process of Scheme 4 simply takes this capability to the molecular range.

Ziegler and co‐workers [29, 30] first reported the “nickel effect” in which a trace amount of nickel was observed to catalyze dimerization of ethene to 1‐butene via competitive “displacement” of the n‐butyl groups on aluminium that are formed upon ethene insertion into the Al‐C bonds of AlEt_3_. While colloidal Ni was determined to be the best catalyst for this process, other Ni sources have appeared in the literature as catalysts for similar transformations. Hence, in their seminal report of iron‐catalyzed coordinative chain transfer polymerization of ethene using stoichiometric excess equivalents of ZnEt_2_ as a chain transfer agent, Gibson and co‐workers [31, 32] reported that, after chain growth and removal (deactivation) of the iron catalyst, treatment of the initially‐formed dialkylzinc product in solution with 5 mol% of Ni(II)bis(acetylacetonate) [Ni(acac)_2_] under ethene pressure served to effect displacement to provide a narrow Poisson distribution (*Ð* = 1.1) of a 9: 1 mixture of linear alkenes and the corresponding saturated linear alkanes with *M*_n_ = 800 Da. However, to the best that we have been able to ascertain, these authors never established the isomeric purity of the isolated alkenes product. Kempe and co‐workers [33, 34] further reported the use of Ni(0)bis(1,5‐cyclooctadiene) [Ni(cod)_2_] within a dual titanium‐mediated catalytic chain transfer cycle that regenerates AlEt_3_ in situ as the CTA while LAOs are continuously being produced. By using a high concentration of AlEt_3_ and low pressure of ethene (e.g. 2 bar), a LAO product of relatively narrow molar mass dispersity (*Ð* = 1.5) centered at *M*_n_ = 260 Da could be obtained. These authors additionally verified the isomeric purity of the product for having the 1‐alkene structure using NMR spectroscopy. It must be noted here that the mechanism by which the Ni‐catalyzed displacement reactions proceed has still not been determined by the authors of these reports. Other investigations of the Ni effect have proposed a wide range of organometallic species, including those with Ni(0), Ni(I), and Ni(II) oxidation states within both mononuclear and bimetallic complexes with different modes of transition‐metal and main‐group‐metal bonding interactions [35].

For our purpose, we recognized from the outset that a displacement catalyst had to be introduced in a manner that would simultaneously kill the ability of the telomerization system to engage in any additional migratory insertions of either ethene or of the desired ω‐substituted α‐olefin product once formed in order to avoid generation of a complex mixture. In addition, it is desirable if the ω‐substituted α‐olefin product can be obtained with high telogen chain efficiency and without formation of high molecular weight polyethene (PE).

In practice, we settled on repeating our original LTCCT experiment using 190 equivalents of the tris(ω‐substituted alkyl) aluminum telogens **1** and 10 equivalents of ZnEt_2_ as a chain transfer mediator relative to the active ion pair initiator **V** generated from one equivalent of **2** with one equivalent of **B** in toluene solution at 50°C under a low pressure of ethene (0.34 bar) [36]. As previously noted, we recognized that a very small amount (ca 3%) of LAO product would be co‐produced from the ZnEt_2_ used as the chain transfer mediator and from the single Me group contributed by the initiator. After 3 h of chain growth, the trialkylaluminum intermediate was subjected to varying mole percent equivalents (relative to total moles of Al + Zn) of either Ni(acac)_2_ or Ni(cod)_2_ as pre‐catalysts for displacement that was conducted in the presence of ethene and at 50°C for an additional 45 min. After isolation and purification using a short silica gel column and hexanes as the eluent, the product was characterized by high field ^1^H and ^13^C NMR (400 and 100 MHz, respectively, CDCl_3_, 25°C) and gas chromatography / mass spectrometry (GC‐MS) (see SI for experimental details).

Figure 1 presents the ^1^H NMR and GC‐MS data for the hydrocarbon product **3a** obtained from LTCCT of telogen **1a** with ethene in which 5 mol% of Ni(acac)_2_ was employed for the displacement step. As can be seen, these data reveal that a mixture of terminal and internal alkenes exist along with a small amount of the saturated H‐terminated alkane product [36]. Although this initial attempt was promising with respect to the high yield of **3a** that was obtained in the absence of any significant formation of PE, the presence of internal alkenes rendered the product unsuitable for consideration as a monomer for coordination polymerization. We also considered employing recently reported methods for catalytic contra‐thermodynamic isomerization to convert the mixture into the desired 1‐alkene isomer—with or without trapping by hydrosilylation or hydroboration [37, 38], but felt this strategy would take us away from achieving the desired initial goal.

**FIGURE 1:**
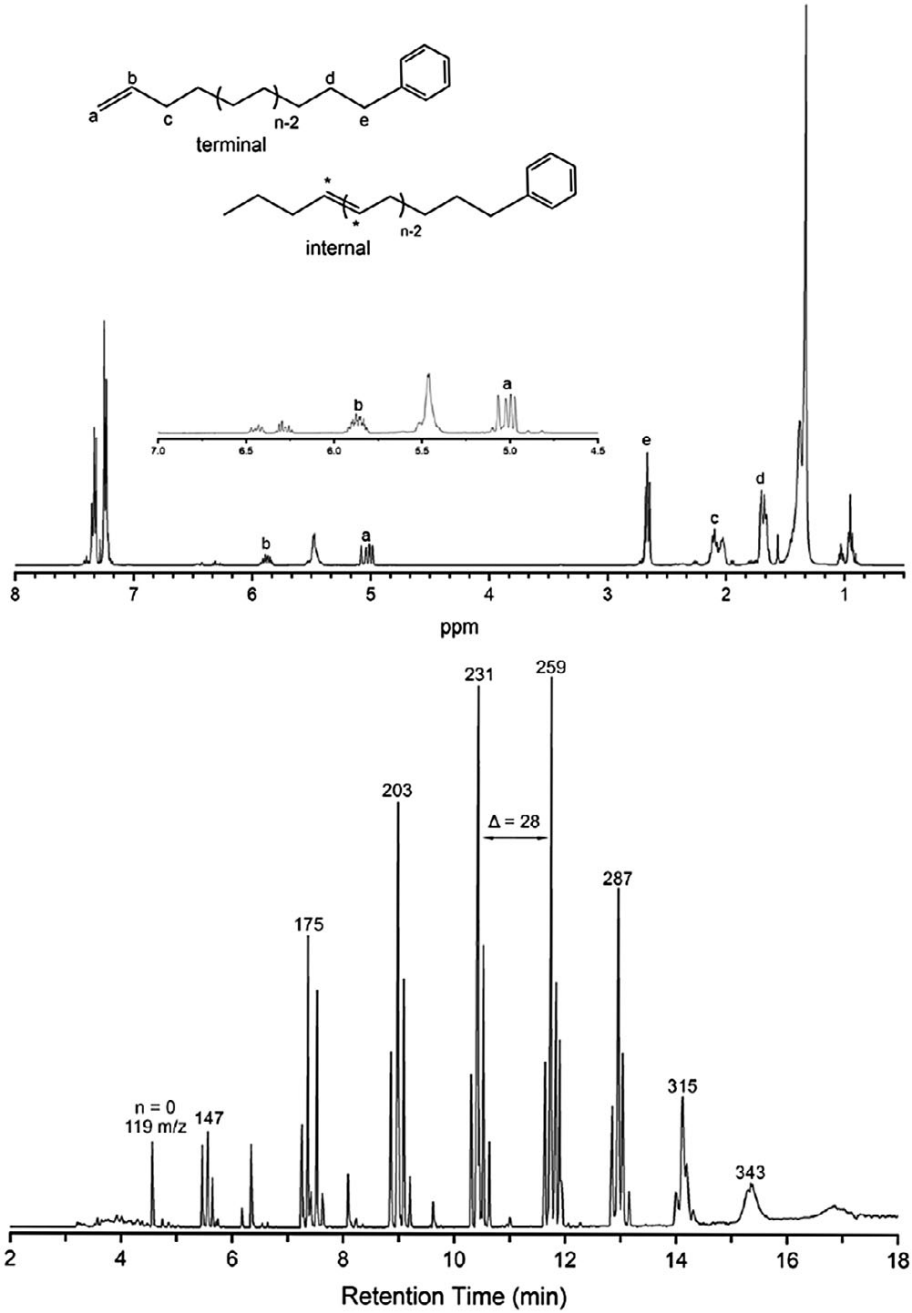
(top) ^1^H NMR (400 MHz, CDCl_3_, 25°C) spectrum and (bottom) GC‐MS of **3a** obtained using 5 mol% Ni(acac)_2_ for displacement step. The resonance marked with an asterisk (*) is assigned to internal alkenes of unknown specific position.

Investigations of the Ziegler Ni‐effect confirm that alkene isomerization can be expected with Ni(II) and that the principal catalytically active species are proposed to be in the Ni(0) oxidation state [35]. Accordingly, we next turned to using Ni(cod)_2_ as the pre‐catalyst for alkyl group displacement of the trialkylaluminum intermediate, and as the ^1^H NMR and GC‐MS data of Figures 2 and 3 respectively confirm, this change was met with substantial success as the isomerically pure, ω‐substituted 1‐alkenes **3a—c** could now be obtained in high isolated yield (e.g. > 65%) through the living telomerization of the corresponding telogens **1a—d** when 5 mol% of Ni(cod)_2_ was employed for the second displacement step (see Figures S13 and S14 for corresponding **3d** data). As expected, a very small secondary *n*‐mer distribution is observed by GC / MS in each derivative of **3** and this can be assigned to the series of LAOs arising from the amount of ZnEt_2_ initially employed as the chain transfer mediator (see peaks marked with asterisk in Figure 3). Further, we have found that various amounts of another small *n*‐mer series for H‐terminated ω‐substituted alkanes arise (see peaks marked with a plus (+) in Figure 3). We have not yet determined the optimum (minimum) time required for completion of the Ni‐catalyzed displacement reaction for each derivative of **3**, and it is possible that a small amount of organoaluminum species is still present upon hydrolysis of the reaction mixture as the source of this n‐alkane product. However, most importantly, ^1^H NMR spectra confirm that isomeric purity for the 1‐alkene product in all derivatives of **3** is at least 99% or better. Gratifyingly, similar results were obtained when the one‐pot, two‐step process was conducted on larger scale using **1a** as the telogen to provide 7.9 g of **3a** and with only a small amount of insoluble PE being formed. Finally, we have been able to demonstrate that nearly identical results can be obtained when the amount of Ni(cod)_2_ employed is reduced to only 0.1 mol% (relative to total moles of CTA employed) [36].

**FIGURE 2:**
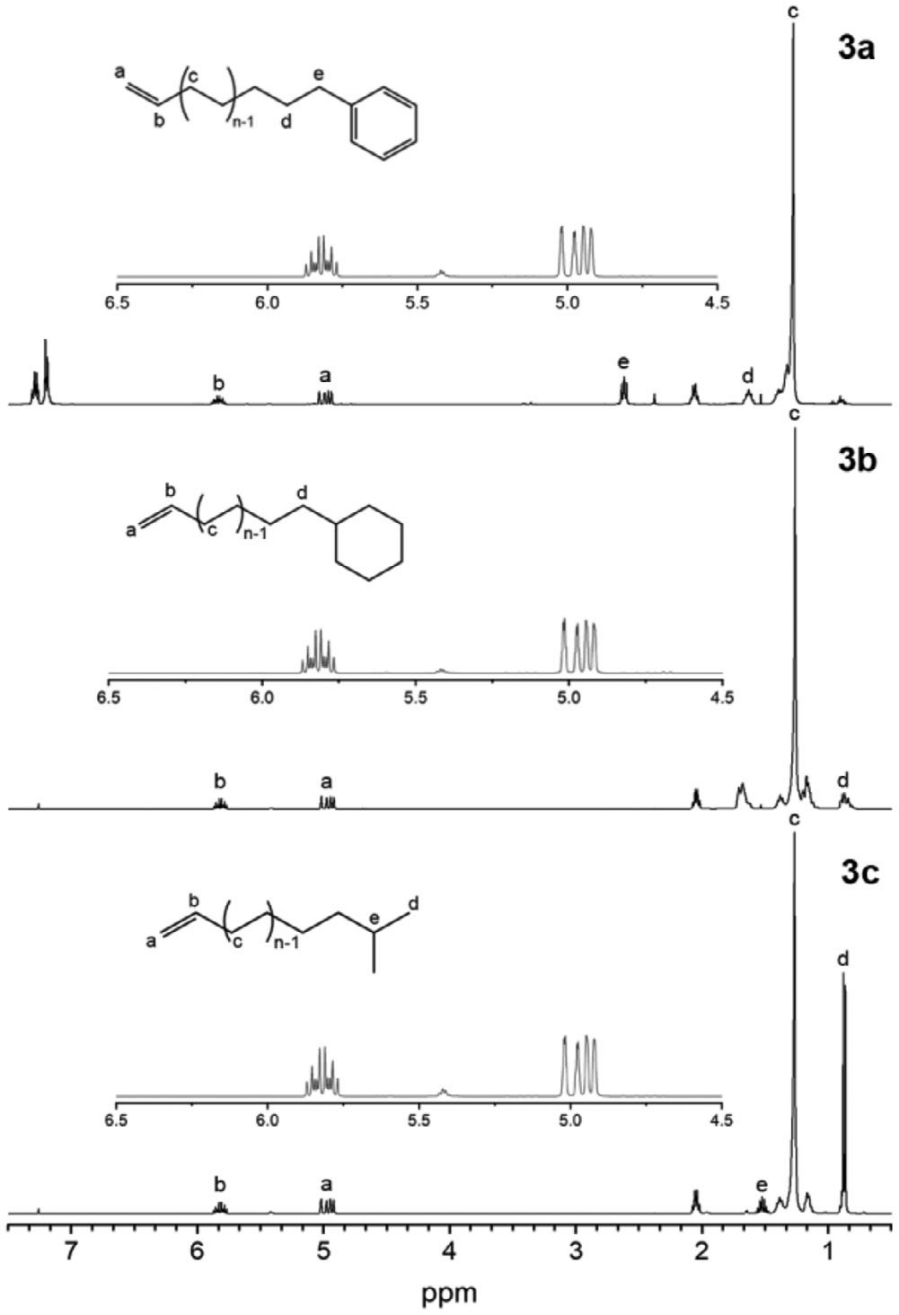
^1^H NMR (400 MHz, CDCl_3_, 25°C) spectra of (top to bottom) **3a**, **3b**, and **3c**.

**FIGURE 3:**
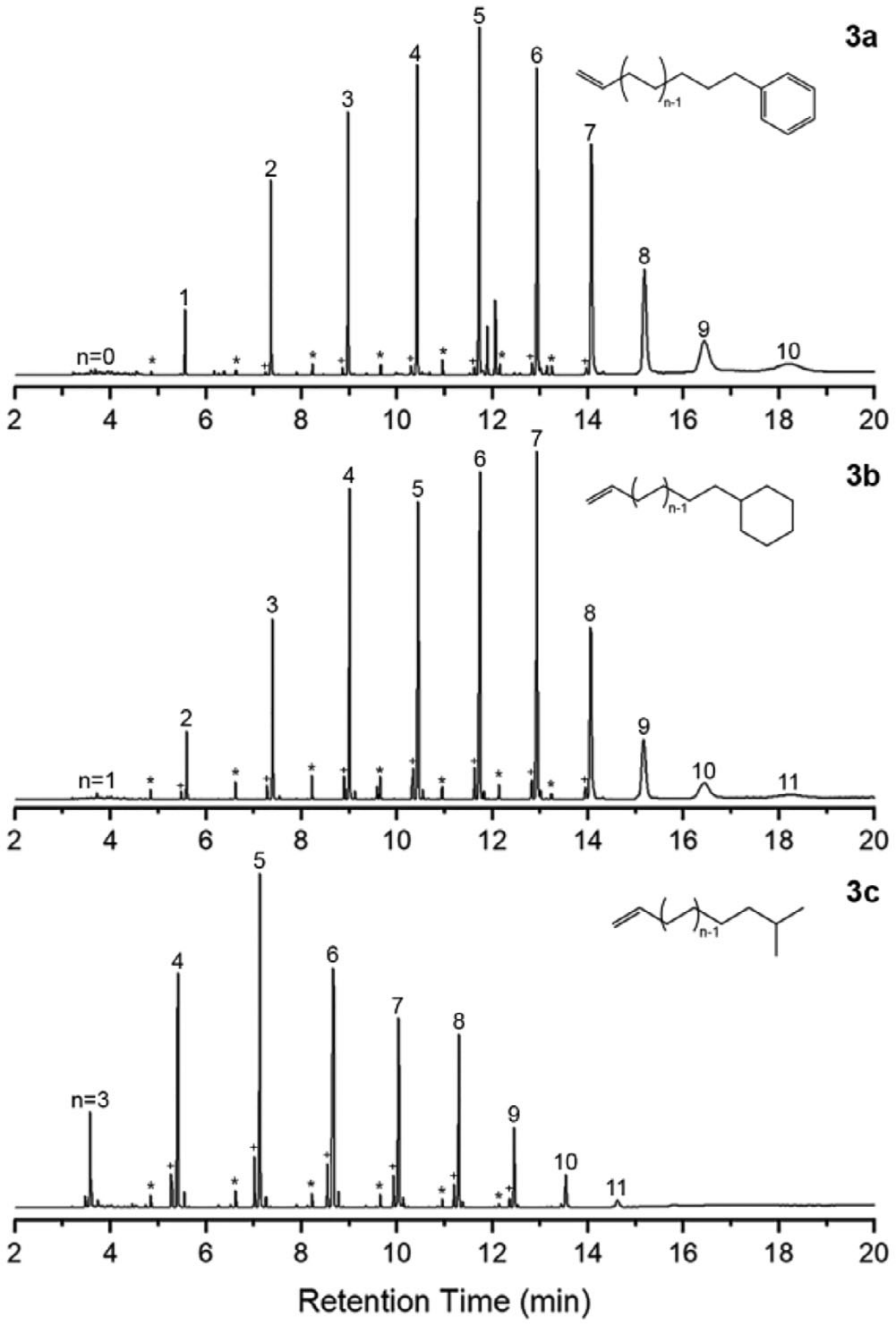
GC‐MS traces for (top to bottom) **3a**, **3b**, and **3c**. The LAO derived from the small amount of ZnEt_2_ used as CTM are marked with an asterisk (*) and saturated hydrocarbons with a plus (+).

As previously stated, the mechanism by which the nickel effect operates with trialkylaluminum and Ni(0) species has remained a long‐enduring mystery [35]. The fact that the process of Scheme 2 remains highly efficient even at very low concentrations of nickel suggests that heterogenous colloidal material, and perhaps even photolytic pathways, might very well be involved. Accordingly, deducing a plausible mechanism for the observed displacement reaction lies outside the scope of the present work. On the other hand, having now established secure access to readily available and scalable quantities of different ω‐substituted 1‐alkenes, we are very interested in exploring the utility and potential of **3** as monomers and comonomers for next generation polyolefins in which new physical properties might emerge and be tuneable through both the nature and level of incorporation of different ω groups.

Scheme 5 presents a summary of new polyolefins that have been prepared from the ω‐substituted α‐olefins **3** when used as monomers for stereoselective living coordination polymerization (LCP) or copolymerization with various α‐olefins using either *C*_s_‐symmetric **2** / **B** for atactic propagation or the *C*_1_‐symmetric CPAM Zr pre‐initiator **5** [18] in combination with **B** for isotactic propagation [29, 30]. In each case, the monomer **3** obtained through the telomerization process of Scheme 4 was used with minimal further purification to provide excellent yields of the targeted polyolefin product. Figure 4 presents a selection of ^1^H NMR (400 MHz) spectra and corresponding differential scanning calorimetry (DSC) data obtained for: (a) the isotactic homopolymer of **3a**, (b) the isotactic random copolymer of **3a** with propene, and (c) the isotactic random copolymer of **3d** with 4‐methyl‐1‐pentene (4M1P) (see Supporting Information for complete characterization of these polyolefins). These polyolefins are of interest due to new functionality and physical properties that can be imparted with inclusion of the ω‐substituted α‐olefin comonomer. For instance, we have previously reported that a phenyl group that is introduced at either the terminal end or within the main chain of a polyolefin that is produced through LCP can be used as a site for new chemical functionality via post aromatic electrophilic substitution reactions [39, 40]. We have also focused attention on commercially significant isotactic poly(4‐methyl‐1‐pentene) (iP4M1P), which is a high melting thermoplastic used for microwave ware and optical lenses, to name a few applications [41]. However, highly stereoregular iP4M1P is intrinsically brittle with low tensile strength, and accordingly, we have been interested in manipulating these and other of its physical properties through programmable introduction of stereoerrors [42, 43], or in this work, through introduction of varying amounts of **3d**, which, interestingly, is also derived from 4M1P via our living telomerization process and telogen **1d** (see Scheme 3). Figure 4c confirms that the *T*_m_ of iP4M1P can be manipulated through variations in the mole percent of incorporated **3d**, which was determined through integration of relevant ^13^C NMR resonances obtained using inverse‐gated ^1^H decoupling. The appearance of multiple melting endotherms, such as that presented by the 2.5% copolymer is a common feature of iP4M1P homopolymer and copolymers and is likely due to formation of different lamellae size and thickness upon crystallization [42, 43]. We are currently investigating physical properties of this family of next generation polyolefins. Finally, Scheme 6 presents the results of incorporation of **3a** as a comonomer with 1,5‐hexadiene that is known to undergo cyclopolymerization during coordination polymerization to provide poly(methylene‐1,3‐cyclopentane) (PMCP) [19, 23, 44]. With **2** as pre‐initiator, the PMCP copolymer with **3a** was produced with a 1,3‐*cis*:1,3‐*trans* ratio of 60:40 for the 1,3‐cyclopentane repeat unit. This *cis*/*trans* ratio was established using previously reported ^13^C NMR resonance assignments [44]. More interestingly, enantioselective LCP with the enantiomerically and diastereomerically‐pure CPAM Hf pre‐initiator **6**, was conducted to provide the optically active copolymer of PMCP with **3a** as comonomer with a 1,3‐*trans*: 1,3‐*cis* ratio of 70:30 ([α]^25^_D_ = (‐)10.9°, *c* = 10 mg/mL, CHCl_3_) [45, 46, 47]. It is well‐known, and we have previously documented, that the 1,3‐*cis* / 1,3‐*trans* ratio in PMCP is highly sensitive to the magnitude of different nonbonded steric interactions of the supporting ligand environment about the transition‐metal center of the propagating species. Although now investigated both experimentally and computationally, this ratio remains an intrinsic feature of different coordination polymerization systems, and it is neither possible to predict nor control.

**SCHEME 5:**
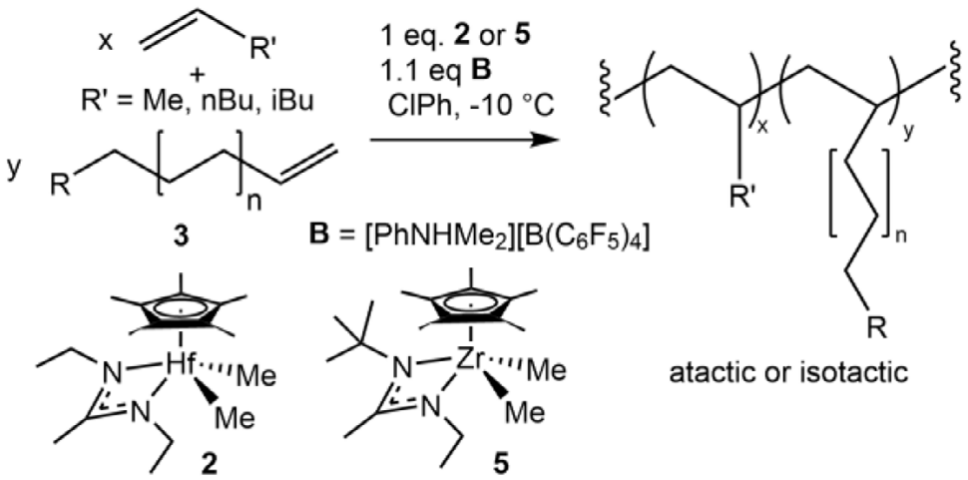
Living coordination (co)polymerizations of various.

**FIGURE 4:**
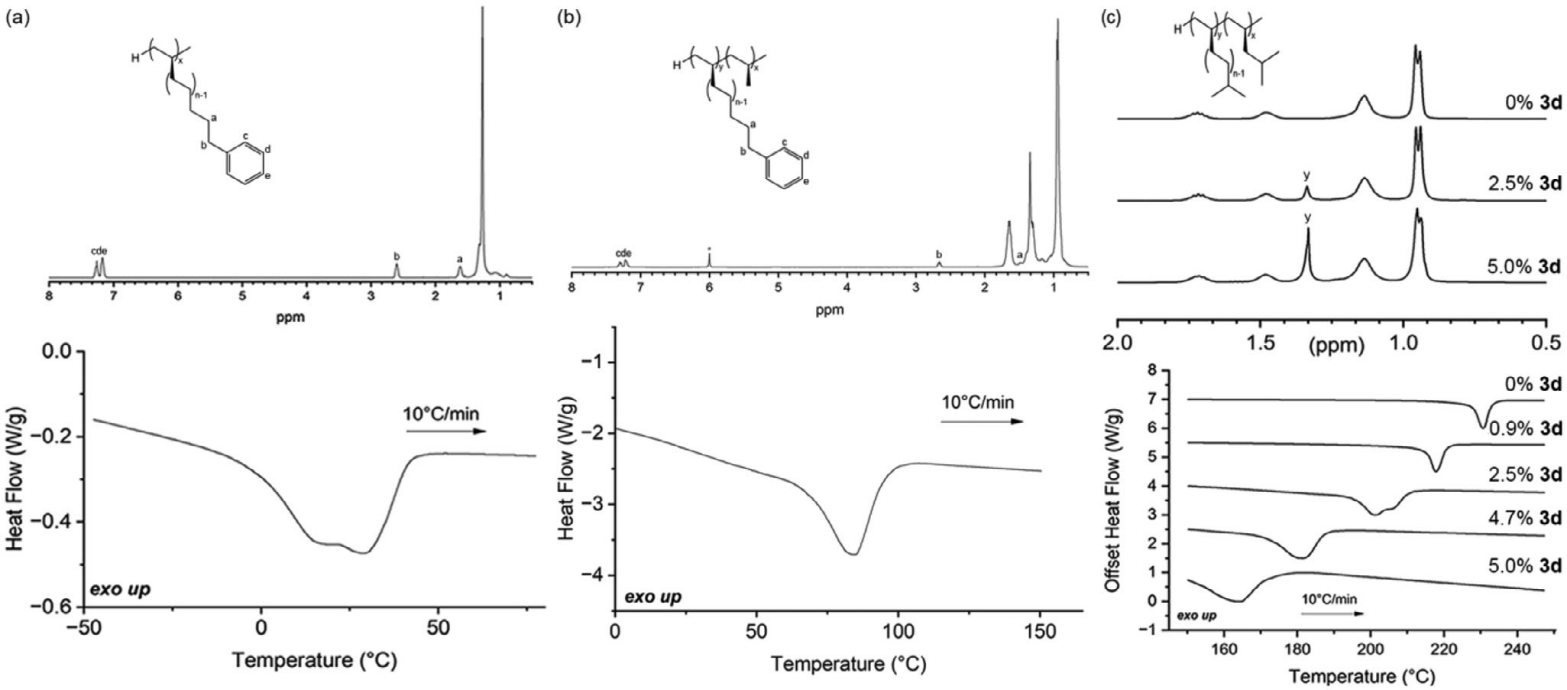
Partial characterization data for homopolymers and copolymers with (top) ^1^H NMR (400 MHz) and (bottom) DSC (second heat ramp, 10°C/min) for (a) isotactic homopolymer of **3a**, (b) isotactic copolymer of propene and **3a**, (c) and isotactic copolymers of 4‐methyl‐1‐pentene (4M1P) with varying levels of incorporation of **3d**. Solvent peak in (b) is marked with an asterisk (*).

**SCHEME 6:**
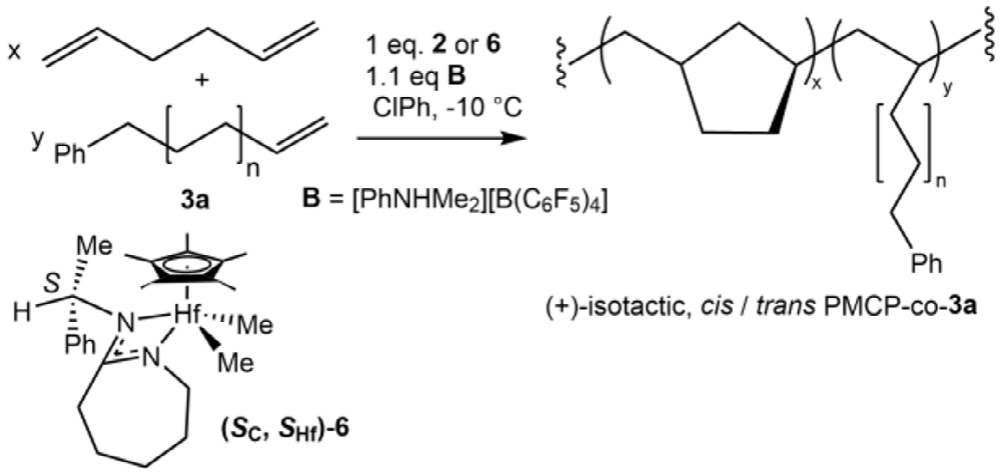
Enantioselective living coordination copolymerization of 1,5‐hexadiene with **3a** as comonomer.

In summary, the present report details a practical and scalable synthesis of a versatile range of new families of ω‐substituted α‐olefins that can be incorporated as next generation polyolefins with increased functionality and tunable physical properties. Current efforts are in progress to provide pure *n*‐mer fractions of **3** via conventional methods and to expand the range of synthetic polymers and materials that these new feedstocks can now provide access to.

## Author Contributions

L.R.S supervised the overall project and wrote the preliminary manuscript. All authors contributed to the analysis, figures, and writing of the final manuscript.

## Conflicts of Interest

The corresponding author has a financial interest in the university spin‐out company, Precision Polyolefins, LLC (PPL). This work did not involve any PPL personnel, funding, or other resources and all new intellectual property has been disclosed in accordance with state and federal requirements.

## Experimental Section

Complete experimental details are provided in the Supporting Information.

## Supporting information




**Supporting File**: anie74058‐sup‐0001‐SuppMat.docx.

## Data Availability

The data that support the findings of this study are available from the corresponding author upon reasonable request.
