# Supplementary material for: Scalable ω‐Substituted α‐Olefins via Living Chain Transfer Telomerization: A Platform for Next Generation Polyolefins
Source: Angew Chem Int Ed Engl. 2026 Aug 7;65(40):e6963058. doi: 10.1002/anie.6963058 (PMC13618262; doi:10.1002/anie.6963058)
Supplement: Supplementary file 1 [file ANIE-65-e6963058-s001.docx]

Supporting Information for the Article Entitled

**Scalable ω-Substituted α-Olefins via Living Chain Transfer Telomerization: A Platform for Next Generation Polyolefins**

Authored by

Lauren G. Logue, Cole M. Burrows, Vince J. Wedekind, Hyunku C. Kim, Jr., and Lawrence R. Sita*

Department of Chemistry and Biochemistry

University of Maryland, College Park, Maryland 20742, USA

*corresponding author email: [lsita@umd.edu](mailto:lsita@umd.edu)

Experimental Details and Supporting Characterization Data.

**Table of Contents**

**General Considerations	S3**

**Experimental Details	S4**

**Supporting NMR Spectra	S11**

**GC-MS Traces	S33**

**^1^H 2D COSY Spectra	S40**

**Supporting DSC Traces	S41**

**Supporting TGA Traces	_ S52**

**Supporting SEC Traces	S63**

**References	S71**

**General Considerations**

All manipulations with air and moisture sensitive materials were carried out under and atmosphere of dinitrogen (N_2_) using standard Schlenk or glovebox techniques. Chlorobenzene was purchased from Millipore Sigma (*ReagentPlus®, 99%*) and dried over calcium hydride by refluxing at 130 °C for three days and distilled under d prior to use. Chloroform was dried over calcium hydride by refluxing at 65 °C for 2 days and distilled under N_2_ prior to use. Toluene (Fisher Scientific, 99%) was dried and deoxygenated by passage over activated alumina and activated copper catalyst (Q5, GetterMax 135®) and collected under N_2_ prior to use. Hexane (Fisher Scientific, HPLC grade) was used as received. Chloroform-*d_1_* was purchased from Cambridge Isotopes and used as received. 1,1,2,2-Tetrachloroethane-*d*_2_ (TCIE-*d*_2_) was purchased from Millipore Sigma and used as received. Bis(1,5-cyclooctadiene)nickel(0) (Ni(cod)_2_), and diethylzinc (ZnEt_2_) were purchased from Sigma-Aldrich and used as received. Nickel(II) bis(acetylacetonate) (Ni(acac)_2_) was obtained from ACROS Organics and used as received. Polymer purity ethylene was purchased from Matheson Trigas, and passed through activated Q5 and molecular sieves (4 Å). Trialkyl aluminum chain transfer agents (CTA) were prepared according to previous literature.^1^ Pre-initiators used in this study include: (η^5^-C_5_Me_5_)[κ^2^-(*N,N*’)-N(Et)C(Me)N(Et)]Hf(Me)_2_(**2**)^2^, (η^5^-C_5_Me_5_)[κ^2^-(*N,N*’)-N(Et)C(Me)N(iBu)]Zr(Me)_2_ (**5**)^3^, and (*S*_C_, *S*_Hf_)-(η^5^-C_5_Me_5_) [(κ^2^-(*N,N*’)-N-(-)-(*S*)-1-phenylethyl)(NimcapN)]-(*S*)-Hf(Me)_2_ (**6**)^4^ were prepared according to previously reported procedures. [PhNHMe_2_][B(C_6_F_5_)_4_] (**B**) was purchased from Ambeed and used without further purification.

**Nuclear Magnetic Resonance (NMR).** ^1^H, ^13^C{^1^H-broadband} and ^13^C{^1^H-inverse gated} NMR spectroscopy was performed on a Bruker AV III 400 MHz or a Bruker AVIII-HD 800 MHz spectrometer fitted with a cryo-QCI probe. Spectra were referenced to residual solvent peaks (^1^H: 7.26 ppm for chloroform-*d*_1_*,* and 6.0 ppm for TCIE-*d*_2_; ^13^C {^1^H}: 77.16 ppm for chloroform-*d*_1_*,* and 73.78 ppm for TCIE-*d*_2_.)

**Gas Chromatography Mass Spectrometry (GC-MS).** The gas chromatography measurements were performed on an Agilent 6890N system coupled with a JEOL high resolution magnetic sector mass spectrometer (JMS-700 MStation) with the EI ion source (70 eV). The mass spectrometer was operated in the mode of high scan speed and low resolution (1000) with the mass range from 40 to 500 Da. The silica capillary column (Agilent DB-17MS, 30 m length, 250 μm I.D.) was used in experiments with helium (at 1 mL/min) as the carrier gas. Analysis was performed as follows: the column temperature was programmed from 70 °C at 2.0 min, increased to 300 °C at the rate of 16 °C/min and held at 300 °C for another 3.62 min. The fragmentation patterns obtained by EI were used to identify the structures of ions observed in the mass spectra.

**Optical Rotation.** Recorded at ambient temperature with a JASCO P-1010 Polarimeter equipped with 589 nm sodium lamp. Solutions were at a fixed concentration of 100mg/10mL with reagent grade chloroform.

**Differential Scanning Calorimetry (DSC).** DSC performed on a TA Instruments Discovery DSC-2500 in T-Zero aluminum sample pans using a heat/cool/heat cycle with a heating rate of 10 °C/min and a cooling rate of 5 °C/min. Data presented is for the second heat cycle and first cool cycle.

**Thermogravimetric Analysis (TGA).** TGA performed on TA Instruments Discovery TGA-5500 in platinum HT sample pans. Samples were heated from 20 °C up to 800 °C at a rate of 20°C/minute.

**High Temperature-Gel Permeation Chromatography (HT-SEC).** A Viscotek HT-GPC Module 350A paired with a Viscotek model 430 heating stage was used with three Tosoh HT columns. 1,2,4-trichlorobenzene (TCB) was used as eluent at 1mL/min and heated at 145 °C. Approximately 40 mg of dry polymer was dissolved in 10mL of TCB while stirring on the heating stage until fully dissolved. Polymer molecular weight indices were obtained relative to broad and narrow polystyrene standards using a triple-detector (refractive index, viscometry, light-scattering) calibration. TCB was filtered through a 0.2-micron filter prior to use.

**Size Exclusion Chromatography (SEC).** A Viscotek GPCMax equipped with two Agilent Technologies PLgel 5 μm MIXED-D columns in a column oven and differential refractometer (Viscotek TDA 302) maintained at 40°C. Tetrahydrofuran (HPLC Grade) was used as the eluent with a flow rate of 1 mL/min. Polymer molecular weight indices were obtained relative to polystyrene standards (1.2 to 215kDa). For room temperature SEC sample preparation, approximately 8 mg of dry polymer sample was dissolved in 2 mL of THF (HPLC Grade).

**Experimental Details**

**General procedure for LTCCT**: In a 100-mL 3-neck round bottom flask equipped with a gas adapter, glass septa and thermometer, a toluene solution of ZnEt_2_ and telogen **1** was added. A toluene solution (2 mL) of 1 equiv. of pre-initiator **2** and 1 equiv. of co-initiator **B** was then added and allowed to stir for 10 minutes. The reaction mixture was then pressurized to 5 psi (0.34 bar) with ethene, and the temperature was slowly raised to 50 °C with external heating while the pressure was maintained for 3 hours with stirring. The ethene was turned off, and a solution of the nickel compound in toluene was added at 50 °C. The reaction mixture was then repressurized to 5 psi with ethene and allowed to stir for 45 minutes. The reaction was slowly quenched with addition of a 10% HCl/MeOH solution and stirred for 30 minutes, after which solids were filtered off. Volatiles were removed in vacuo to yield the crude ω-substituted 1-alkene product, which was then subjected to purification by dissolving into a minimum amount of hexanes and passing this through a silica gel (400 mesh) and removal of the volatiles to provide a white material. Specific details for the synthesis of ω-substituted 1-alkenes are listed below.

**Synthesis of ω-phenyl substituted 1-alkene 3a_1_ using 5 mol% of Ni(acac)_2_.**

The general procedure was followed using (a) a solution of 1.7 g (4.4 mmol) of tri (3-phenyl propyl) aluminum (**1a**) and 29 mg (0.23 mmol) of ZnEt2 in 29 mL of toluene, followed by addition of the initiator from 11 mg (23 μmol) of **2** and 19 mg (23 μmol) of **B** in toluene and stirred for 3 hours at 50 °C before addition of a solution of 60 mg (0.24 mmol) of Ni(acac)2, in 5 mL of toluene to the flask. The mixture was monitored by ^1^H NMR aliquots that showed the reaction to be complete in 45 minutes after the addition of Ni(acac)2. The reaction was quenched and the product purified according to the general procedure to provide 1.4 g of **3a_1_**. ^1^H NMR and ^13^C{^1^H} NMR spectra for 3a are shown in Figures S1 and S2 respectively. ^1^H NMR (400 MHz, chloroform-*d*): 1.33 (c, 13H, m), 1.64 (d, 3H, m), 2.62 (e, 2H, t, *J* = 7.8 Hz), 4.99 (a, 0.7H, m), 5.83 (b, 0.35H, m) 7.20 (3H, m), 7.30 (2H, m). ^13^C NMR (100 MHz, chloroform-*d*_1_): 114 (b), 126 (f), 128 (e), 129 (d), 140 (a), 143 (c). GC-MS traces and ^1^H 2D COSY spectra for **3a_1_** are shown in Figures S45 and S52. DSC and TGA results can be found in Table 1.2.

**Synthesis of ω-phenyl substituted 1-alkene 3a_2_ using 5 mol% of Ni(cod)_2_.**

The general procedure was performed using the following amounts of reagents: 11 mg (23 μmol) of **2**, 19 mg (23 μmol) of **B**, 1.7 g (4.4 mmol) of tri (3-phenyl propyl) aluminum (**1a**), 29 mg (0.23 mmol) of ZnEt2, 29 mL toluene and 65 mg (0.24 mmol) of Ni(cod)2, in 5 mL toluene. The reaction was complete in 45 minutes and was quenched and product purified according to the general procedure above to provide 2.9 g of **3a_2_**. ^1^H NMR and ^13^C{^1^H} NMR spectra are shown in Figures S3 and S4, respectively. ^1^H NMR (400 MHz, chloroform-*d*): 1.33 (c, 23H, m), 1.65 (d, 2H, p, *J* = 7.3 Hz), 2.06 (2H, q, *J* = 6.9 Hz), 2.62 (e, 2H, t, *J* = 7.8 Hz), 4.98 (a, 2H, m), 5.84 (b, 1H, m) 7.20 (3H, m), 7.28 (2H, m). ^13^C NMR (100 MHz, chloroform-*d*): 114 (b), 126 (f), 128 (e), 129 (d), 139 (a), 143 (c). GC-MS trace is shown in Figure S46. DSC and TGA results can be found in Table 1.1.

**Synthesis of ω-phenyl substituted 1-alkenes 3a_3_ with 1 mol% Ni(cod)_2_.**

The general procedure was performed using the followed amounts of reagents: 11 mg (23 μmol) of **2**, 19 mg (23 μmol) of **B**, 1.7 g (4.4 mmol) of **1a**, 29 mg (0.23 mmol) of ZnEt2 in 29 mL toluene and 13 mg (47 μmol) Ni(cod)2, in 2 mL toluene. Purification provided 2.1 g of **3a_2_**. ^1^H NMR and ^13^C{^1^H} NMR spectra are shown in Figures S5 and S6, respectively. ^1^H NMR (400 MHz, chloroform-*d*): 1.30 (c, 14H, m), 1.66 (d, 2H, p, *J* = 7.5 Hz), 2.08 (2H, q, *J* = 7.0 Hz), 2.64 (e, 2H, t, *J* = 7.8 Hz), 4.99 (a, 2H, m), 5.86 (b, 1H, m) 7.21 (3H, m), 7.31 (2H, m). ^13^C NMR (100 MHz, chloroform-*d*): 114 (b), 126 (f), 128 (e), 129 (d), 139 (a), 143 (c). GC-MS trace is shown in Figure S47. DSC and TGA results can be found in Table 1.1.

**Synthesis of ω-phenyl substituted 1-alkenes 3a_3_ with 0.1 mol% Ni(cod)_2_.**

The general procedure was performed using the followed amounts of reagents: 11 mg (23 μmol) of **2**, 19 mg (23 μmol) of **B**, 1.7 g (4.4 mmol) of **1a**, 29 mg (0.23 mmol) of ZnEt2, 29 mL toluene and 1.3 mg (4.7 μmol) Ni(cod)2, in 1 mL toluene. Purification provided 1.7 g of **3a_3_**. ^1^H NMR and ^13^C{^1^H} NMR spectra are shown in Figures S7 and S8, respectively. ^1^H NMR (400 MHz, chloroform-*d*): 1.35 (c, 10H, m), 1.68 (d, 2H, p, *J* = 7.3 Hz), 2.11 (2H, q, *J* = 6.7 Hz), 2.66 (e, 2H, t, *J* = 7.9 Hz), 5.03 (a, 2H, m), 5.88 (b, 1H, m) 7.23 (3H, m), 7.33 (2H, m). ^13^C NMR (100 MHz, chloroform-*d*): 114 (b), 126 (f), 128 (e), 129 (d), 139 (a), 143 (c). GC-MS trace is shown in Figure S48. DSC and TGA results can be found in Table 1.1.

**Synthesis of cyclohexyl substituted 1-alkenes 3b_4_ with 5 mol% Ni(cod)_2_:**

The general procedure was performed using the followed amounts of reagents: 9.4 mg (20 μmol) of **2**, 16 mg (20 μmol) of **B**, 1.4 g (3.9 mmol) of tri (2-cyclohexyl ethyl) aluminum (**1b**), 25 mg (0.20 mmol) of ZnEt2, 27 mL toluene and stirred for 3 hours at 50 °C before adding 58 mg (0.21 mmol) Ni(cod)2, in 4 mL toluene. The mixture was monitored by ^1^H NMR and was complete in 45 minutes. Isolated amount of **3b_4_** was 1.7g. ^1^H NMR and ^13^C{^1^H} NMR spectra are shown in Figures S9 and S10, respectively. ^1^H NMR (400 MHz, chloroform-*d*): 0.86 (d, 3H, m), 1.26 (c, 30H, m) 2.04 (2H, q, *J* = 6.9 Hz), 4.96 (a, 2H, m), 5.82 (b, 1H, m). ^13^C NMR (100 MHz, chloroform-*d_1_*): 37.7 (c), 37.9 (d), 114 (a), 139 (b). GC-MS trace is shown in Figure S49. DSC and TGA results can be found in Table 1.2.

**Synthesis of odd-carbon-numbered-ω-*iso*-butyl 1-alkenes 3c**

The general procedure was performed using the followed amounts of reagents: 10 mg (22 μmol) of **2**, 18 mg (22 μmol) of **B**, 1 g (4.2 mmol) of tri (3-methylbutyl) aluminum (**1c**), 27 mg (0.22 mmol) of ZnEt2, 28 mL toluene and stirred for 3 hours at 50 °C before adding 61 mg (0.22 mmol) Ni(cod)2, in 5 mL toluene. The mixture was monitored by ^1^H NMR and was complete in 45 minutes. Isolated amount of **3c** was 1.5 g. ^1^H-NMR and ^13^C{^1^H} NMR spectra are shown in Figures S11 and S12, respectively. ^1^H NMR (400 MHz, chloroform-*d*): 0.87 (d, 6H, m), 1.27 (c, 25H, m) 1.52 (e, 1H, m), 2.05 (2H, q, *J* = 7.2 Hz), 4.96 (a, 2H, m), 5.82 (b, 1H, m). ^13^C NMR (100 MHz, chloroform-*d_1_*): 22.8 (d), 39.3 (c), 114 (a), 139 (b). GC-MS trace shown in Figure S50. DSC and TGA results can be found in Table 1.1.

**Synthesis of even-carbon-numbered-*iso*-butyl 1-alkenes 3d.**

The general procedure was performed using the followed amounts of reagents: 15 mg (32 μmol) of **2**, 25 mg (32 μmol) of **B**, 1.7 g (6.0 mmol) of tri (4-methylpentyl) aluminum (**1d**), 39 mg (0.32 mmol) of ZnEt2, 40 mL toluene and stirred for 3 hours at 50 °C before adding 1.7 mg (6.3 μmol) Ni(cod)2, in 1 mL toluene. The mixture was monitored by ^1^H NMR and was complete in 45 minutes. Isolated amount of **3d** was 3.5 g. ^1^H NMR and ^13^C{^1^H} NMR spectra are shown in Figures S13 and S14, respectively. ^1^H NMR (400 MHz, chloroform-*d*): 0.86 (f, 6H, m), 1.27 (d, 38H, m) 1.51 (e, 1H, m), 2.04 (c, 2H, q, *J* = 7.1 Hz), 4.96 (a, 2H, m), 5.82 (b, 1H, m). ^13^C NMR (100 MHz, chloroform-*d_1_*): 22.7 (d), 39.1 (c), 114 (a), 139 (b). GC-MS trace shown in Figure S51. DSC and TGA results can be found in Table 1.1.

**Table 1.1- DSC and TGA results of ω-substituted 1-alkenes**

| Alkene | Yield (g) | *T*_m_ (°C) ^a^ | *T*_c_ (°C) ^a^ | Decomp Onset (°C) ^b^ |
| --- | --- | --- | --- | --- |
| **3a_1_** | 1.4 | - | - | 138 |
| **3a_2_** | 2.9 | 48.6 | 48.4 | 155 |
| **3a_3_** | 2.1 | 51.6 | 47.9 | 125 |
| **3a_4_** | 1.7 | 200 | - | 112 |
| **3b** | 1.7 | 47.3 | 43.1 | 128 |
| **3c** | 1.5 | 75.6 | 72.6 | 114 |
| **3d** | 3.5 | 57.5, 40.3 | 53.5 | 124 |

^a^Determined by DSC, ^b^Determined by TGA

**General procedure for homopolymerizations of 3a and 3b**

All polymerizations were performed using **3a_2_** and **3b** obtained from using Ni(cod)_2_ for displacement. In a glovebox, a solution of monomer (**3a_2_** or **3b**) in 4 mL of chlorobenzene was passed through a ‘column’ of short silica gel (400 mesh, dried at 250 °C in an oven prior to use) placed within a glass pipette to remove any trace amounts of moisture. To a round-bottom flask was added the solution of monomer and, optionally ZnEt_2_ if LCCTP was to be performed, and the mixture chilled to 0°C. A solution of the pre-initiator (**M**) (1 eq, 20 μmol) in 1 mL of chlorobenzene at 0°C was added to **B** (18 mg, 1.1 eq, 22 μmol) and mixed until a yellow solution resulted, upon which this was then quickly added to the flask containing monomer. The reaction mixture was stirred for 18 h at 0°C and then quenched with excess 10% acidic methanol for runs 1, 2, and 4, or 1.2 equivalents of I_2_ in toluene for run 3. The solvent was removed *in vacuo*. The resulting polymer was taken up in a minimum amount of chloroform and passed through as short silica gel column using chloroform as the eluent. The polymer product was obtained as a white solid upon removing volatiles in vacuo. Each polymer from the different were characterized by SEC, DSC, and TGA. Values for this data are available in Table 1.2. ^1^H and ^13^C {^1^H-inverse gated} NMR spectra are presented in Figures S15-S22.

**Table 1.2-Homopolymerization Results**

| Run | Initiator  (**M**) | Monomer | CTA (eq) | Yield (mg, %) | *M*_n_(kDa) ^a^ | *Đ* ^a^ | *T*_g_ (°C)^b^ | *T*_m_(°C)^b^ | *T*_c_(°C) ^b^ | Decomp.Onset (°C) ^c^ |
| --- | --- | --- | --- | --- | --- | --- | --- | --- | --- | --- |
| 1 | **2** | **3a** | - | 221, 90% | 15.2 | 1.11 | - | 22.5 | 13.0 | 441 |
| 2 | **5** | **3a** | - | 192, 77% | 23.7 | 1.11 | - | 19.3, 28.8 | 16.9 | 445 |
| 3 | **2** | **3a** | ZnEt_2_ (5) | 209, 84% | 621 (Da) | 1.36 | - | 14.6 | 9.8 | 194 |
| 4 | **2** | **3b** | - | 190, 76% | 11.0 | 1.10 | -42.5 | 55.4 | 47.7 | 366 |

^a^ Determined by SEC (THF, 40 °C). ^b^Determined by DSC, ^c^Determined by TGA

**General procedure for copolymerization of 3 with various α-olefins and 1,5-hexadiene**

To a 50-mL round bottom flask, the w-substituted α-olefin **3** (21.1 eq, 0.42 mmol) was added after passing through silica gel plug with 4.5 mL chlorobenzene as the eluent to remove any trace moisture. To a stirring solution of **3** was added the α-olefin comonomer (189 eq, 3.79 mmol), except for runs 7 and 8 in which propene was introduced and maintained at 0.34 bar, at 0°C. A yellow solution of **B** (18 mg, 1.1 eq, 22 μmol) and pre-initiator **M** (1 eq, 20 μmol) in 0.5 mL of chlorobenzene was then quickly added all at once. The reaction mixture was stirred for 18 hours at 0°C before addition of 5 mL of acidic methanol (10% HCl). The crude product was isolated by removal of all volatiles and then purified by taking it up in 10 mL of toluene and reprecipitating through addition to 100 mL methanol. After filtration and drying under vacuum, the purified polymer was obtained as a white solid. The exception was that the crude product from Run 5 was not purified by reprecipitation, but instead through passing a solution through a short silica gel column using 300 mL of a 20:80 chloroform/hexanes mixture as eluent. All polymers were characterized by HT-SEC or SEC, DSC, and TGA. Values for this data are available in Table 1.3. ^1^H and ^13^C{^1^H-inverse gated} NMR spectra can be found in Figures S23-47.

**Table 1.3-Copolymerization results from LCP of Monomer A and B.**

| Run | Initiator  (**M**) | Monomer A | Monomer B | Yield (mg, %) | Percent  Incorp. (%) | *M*_n_(kDa) ^a^ | *Đ* ^a^ | *T*_g_ ^b^ | *T*_m_(°C) ^b^ | *T*_c_(°C) ^b^ | Decomp.Onset (°C) ^c^ |
| --- | --- | --- | --- | --- | --- | --- | --- | --- | --- | --- | --- |
| 5 | 5 | 1-hex | **3a_2_** | 389, 87% | 8.9 | 56.7 | 1.84 | -21.1 | - | - | 388 |
| 6 | 5 | 4M1P | **3a_2_** | 421, 84% | 8.3 | 132 | 1.05 | -14.8 | 124 | 82.1 | 356 |
| 7 | 5 | Propene | **3a_2_** | 121* | 2.8 | 35.7 | 1.43 | -26.4 | 84.7 | 45.4 | 360 |
| 8 | 2 | Propene | **3a_2_** | 2485* | 4.5 | 249 | 1.39 | -3.26 | - | - | 391 |
| 9 | 2 | 1,5-HD | **3a_2_** | 299, 75% | 10 | 27.3 | 1.65 | -25.3 | 42.8 | - | 453 |
| 10 | 6 | 1,5-HD | **3a_2_** | 658, 82% | 6.8 | 13.4 | 2.37 | -24.0 | 0.3, 36.7 | -8.5 | 459 |
| 11 | 5 | 4M1P | **3c** | 286, 92% | 9.9 | 124 | 1.06 | -6.36 | 124 | 85.6 | 365 |
| 12 | 5 | 4M1P | **3d** | 652, 65% | 0.9 | 662 | 1.29 | - | 218 | 197 | 327 |
| 13 | 5 | 4M1P | **3d** | 615, 62% | 2.5 | 641 | 1.35 | 15.6, 47.9 | 201, 206 | 181 | 314 |
| 14 | 5 | 4M1P | **3d** | 611, 61% | 4.7 | 733 | 1.32 | -2.56, 20.9 | 181 | 155 | 346 |
| 15 | 5 | 4M1P | **3d** | 714, 71% | 5.0 | 1173 | 1.33 | -2.64, 49.9 | 164 | 130 | 345 |

^a^ Run 4 and 8 determined by SEC (THF, 40 °C), runs 5, 6, 7, and 9-15 employed HT-SEC (1,2,4 trichlorobenzene, 145 °C). ^b^Determined by DSC, ^c^Determined by TGA. Run **10** had a specific optical rotation recorded at room temperature: [α]^25^_D_ = –10.9° (c = 100 mg/10mL, CHCl_3_)

**^1^H NMR and ^13^C{^1^H} NMR for 3 and ^13^C{^1^H-inverse gated} NMR spectra for polyolefins**

**Figure S1:** ^1^H NMR spectrum of **3a_1_** (400 MHz, 25 °C, CDCl_3_ (*) )

**Figure S2:** ^13^C{^1^H} NMR spectrum of **3a_1_** (100 MHz, 25 °C, CDCl_3_ (*))

**Figure S3:** ^1^H NMR spectrum of **3a_2_** (400 MHz, 25 °C, CDCl_3_ (*))

**Figure S4:** ^13^C{^1^H} NMR spectrum of **3a_2_** (100 MHz, 25 °C, CDCl_3_ (*))

**Figure S5:** ^1^H NMR spectrum of **3a_3_** (400 MHz, 25 °C, CDCl_3_ (*))

**Figure S6:** ^13^C{^1^H} NMR spectrum of **3a_3_** (100 MHz, 25 °C, CDCl_3_ (*))

**Figure S7:** ^1^H NMR spectrum of **3a_4_** (400 MHz, 25 °C, CDCl_3_ (*))

**Figure S8:** ^13^C{^1^H} NMR spectrum of **3a_4_** (100 MHz, 25 °C, CDCl_3_ (*))

**Figure S9:** ^1^H NMR spectrum of **3b** (400 MHz, 25 °C, CDCl_3_ (*))

**Figure S10** ^13^C{^1^H} NMR spectrum of **3b** (100 MHz, 25 °C, CDCl_3_ (*))

**Figure S11:** ^1^H NMR spectrum of **3c** (400 MHz, 25 °C, CDCl_3_ (*))

.

**Figure S12:** ^13^C{^1^H} NMR spectrum of **3c** (100 MHz, 25 °C, CDCl_3_ (*))

**Figure S13:** ^1^H NMR spectrum of **3d** (400 MHz, 25 °C, CDCl_3_ (*))

.

**Figure S14:** ^13^C{^1^H} NMR spectrum of **3d** (100 MHz, 25 °C, CDCl_3_ (*))

**Figure S15:** ^1^H NMR spectrum of **Run 1** (400 MHz, 25 °C, CDCl_3_ (*))

**Figure S16:** ^13^C{^1^H-inverse gated} NMR spectrum of **Run 1** (100 MHz, 25 °C, CDCl_3_ (*))

**Figure S17:** ^1^H NMR spectrum of **Run 2** (400 MHz, 25 °C, CDCl_3_ (*))

**Figure S18:** ^13^C{^1^H-inverse gated} NMR spectrum of **Run 2** (100 MHz, 25 °C, CDCl_3_ (*))

**Figure S19:** ^1^H NMR spectrum of **Run 3** (400 MHz, 25 °C, CDCl_3_ (*))

**Figure S20:** ^13^C{^1^H-inverse gated} NMR spectrum of **Run 3** (100 MHz, 25 °C, CDCl_3_ (*))

**Figure S21:** ^1^H NMR spectrum of **Run 4** (400 MHz, 25 °C, CDCl_3_ (*))

**Figure S22:** ^13^C{^1^H-inverse gated} NMR spectrum of **Run 4** (100 MHz, 25 °C, CDCl_3_ (*))

**Figure S23:** ^1^H NMR spectrum of **Run 5** (400 MHz, 25 °C, CDCl_3_ (*))

**Figure S24:** ^13^C{^1^H-inverse gated} NMR spectrum of **Run 5** (100 MHz, 25 °C, CDCl_3_ (*))

**Figure S25:** ^1^H NMR spectrum of **Run 6** (400 MHz, 90 °C, TCIE-*d*_2_ (*))

**Figure S26:** ^13^C{^1^H-inverse gated} NMR spectrum of **Run 6** (100 MHz, 90 °C, TCIE-*d*_2_ (*))

**Figure S27:** ^1^H NMR spectrum of **Run 7** (400 MHz, 90 °C, TCIE-*d*_2_ (*))

**Figure S28:** ^13^C{^1^H-inverse gated} NMR spectrum of **Run 7** (100 MHz, 90 °C, TCIE-*d*_2_ (*))

**Figure S29:** ^1^H NMR spectrum of **Run 8** (400 MHz, 90°C, TCIE-*d*_2_ (*))

**Figure S30:** ^13^C{^1^H-inverse gated} NMR spectrum of **Run 8** (100 MHz, 90°C, TCIE-*d*_2_ (*))

**Figure S31:** ^1^H NMR spectrum of **Run 9** (400 MHz, 25°C, CDCl_3_ (*)).

**Figure S32:** ^13^C{^1^H-inverse gated} NMR spectrum of **Run 9** (100 MHz, 25°C, CDCl_3_ (*)). Resonances are marked according to assignments previously reported in the literature.^5,6^

**Figure S33:** ^1^H NMR spectrum of **Run 10** (400 MHz, 25°C, CDCl_3_ (*)).

**Figure S34:** ^13^C{^1^H-inverse gated} NMR spectrum of **Run 10** (100 MHz, 25°C, CDCl_3_ (*)).

**Figure S35:** ^1^H NMR spectrum of **Run 11** (400 MHz, 90°C, TCIE-*d*_2_ (*))

[Grab your reader’s attention with a great quote from the document or use this space to emphasize a key point. To place this text box anywhere on the page, just drag it.]

**Figure S36:** ^13^C{^1^H-inverse gated}NMR spectrum of **Run 11** (100 MHz, 90°C, TCIE-*d*_2_ (*))

**Figure S37:** ^1^H NMR spectrum of **Run 12** (400 MHz, 100°C, TCIE-*d*_2_ (*))

**Figure S38:** ^13^C{^1^H-inverse gated} NMR spectrum of **Run 12** (200 MHz, 100°C, TCIE-*d*_2_ (*))

**Figure S39:** ^1^H NMR spectrum of **Run 13** (400 MHz, 90°C, TCIE-*d*_2_ (*))

**Figure S40:** ^13^C{^1^H-inverse gated}NMR spectrum of **Run 13** (200 MHz, 90°C, TCIE-*d*_2_ (*))

**Figure S41:** ^1^H NMR spectrum of **Run 14** (400 MHz, 90°C, acetone(+), TCIE-*d*_2_ (*))

**Figure S42:** ^13^C{^1^H-inverse gated}NMR spectrum of **Run 14** (200 MHz, 90°C, TCIE-*d*_2_ (*))

**Figure S43:** ^1^H NMR spectrum of **Run 15** (400 MHz, 90°C, TCIE-*d*_2_ (*))

**Figure S44:** ^13^C{^1^H-inverse gated}NMR spectrum of **Run 15** (200 MHz, 90°C, TCIE-*d*_2_ (*))

**GC-MS Traces**

**Figure S45:** GC chromatogram of **3a_1_**.

**Table 2.1-Relative %fractions of compound 3a_1_**

| ***n*** | **1-alkene-R** | **H-alkane** | **Int-alkenes** | **1-alkene-Et** |
| --- | --- | --- | --- | --- |
| **0** | 1.42 | N/O | N/O | N/O |
| **1** | 1.80 | 1.09 | 0.75 | N/O |
| **2** | 3.64 | 2.40 | 3.94 | N/O |
| **3** | 8.80 | 2.65 | 4.76 | N/O |
| **4** | 11.67 | 2.89 | 5.76 | N/O |
| **5** | 10.90 | 2.89 | 7.96 | N/O |
| **6** | 7.47 | 2.15 | 3.98 | 1.58 |
| **7** | 3.11 | 0.79 | 0.98 | 0.96 |
| **8** | 2.73 | 0.68 | 0.47 | 0.32 |
| **9** | N/O | N/O | N/O | 0.18 |
| **10** | N/O | N/O | N/O | 0.06 |

**Figure S46:** GC chromatogram of **3a_2_**.

**Table 2.2-Relative %fractions of compound 3a_2_**

| ***n*** | **1-alkene-R** | **H-alkane** | **1-alkene-Et** |
| --- | --- | --- | --- |
| **0** | 0.13 | 0.11 | N/O |
| **1** | 1.99 | 0.07 | N/O |
| **2** | 5.24 | 0.14 | N/O |
| **3** | 7.64 | 0.23 | N/O |
| **4** | 10.86 | 0.33 | N/O |
| **5** | 12.93 | 0.36 | 0.09 |
| **6** | 14.17 | 0.38 | 0.22 |
| **7** | 14.30 | 0.29 | 0.38 |
| **8** | 12.42 | N/O | 0.48 |
| **9** | 8.18 | N/O | 0.37 |
| **10** | 2.67 | N/O | 0.34 |

**Figure S47:** GC chromatogram of **3a_3_**.

**Table 2.3-Relative %fractions of compound 3a_3_**

| ***n*** | **1-alkene-R** | **H-alkane** | **1-alkene-Et** |
| --- | --- | --- | --- |
| **0** | 1.04 | 0.51 | N/O |
| **1** | 4.52 | 0.13 | N/O |
| **2** | 8.26 | 0.21 | N/O |
| **3** | 11.46 | 0.29 | N/O |
| **4** | 13.92 | 0.33 | N/O |
| **5** | 15.01 | 0.31 | 0.37 |
| **6** | 14.21 | 0.17 | 0.49 |
| **7** | 10.66 | N/O | 0.59 |
| **8** | 6.57 | N/O | 0.54 |
| **9** | 3.34 | N/O | 0.41 |
| **10** | N/O | N/O | 0.26 |

**Figure S48:** GC chromatogram of **3a_4_**.

**Table 2.4-Relative %fractions of compound 3a_4_**

| ***n*** | **1-alkene-R** | **H-alkane** | **1-alkene-Et** |
| --- | --- | --- | --- |
| **0** | 3.03 | 2.61 | N/O |
| **1** | 10.75 | 0.81 | N/O |
| **2** | 15.58 | 0.79 | N/O |
| **3** | 16.78 | 0.76 | N/O |
| **4** | 13.88 | 0.65 | N/O |
| **5** | 11.24 | 0.40 | 0.55 |
| **6** | 7.60 | 0.19 | 0.56 |
| **7** | 3.97 | N/O | 0.43 |
| **8** | 1.51 | N/O | 0.23 |
| **9** | 0.02 | N/O | 0.11 |

**Figure S49:** GC chromatogram of **3b**.

**Table 2.5-Relative %fractions of compound 3b**

| ***n*** | **1-alkene-R** | **H-alkane** | **1-alkene-Et** |
| --- | --- | --- | --- |
| **1** | 0.13 | N/O | N/O |
| **2** | 2.03 | 0.22 | N/O |
| **3** | 5.49 | 0.58 | N/O |
| **4** | 10.03 | 0.88 | N/O |
| **5** | 13.54 | 1.15 | 0.29 |
| **6** | 16.89 | 1.12 | 0.54 |
| **7** | 14.81 | 0.96 | 0.71 |
| **8** | 12.49 | 0.55 | 0.55 |
| **9** | 8.87 | N/O | 0.60 |
| **10** | 4.25 | N/O | 0.49 |
| **11** | 1.50 | N/O | N/O |

**Figure S50:** GC chromatogram of **3c**.

**Table 2.6-Relative %fractions of compound 3c**

| ***n*** | **1-alkene-R** | **H-alkane** | **Int-alkenes** | **1-alkene-Et** |
| --- | --- | --- | --- | --- |
| **3** | 4.29 | 0.62 | 0.23 | N/O |
| **4** | 15.71 | 1.78 | 0.67 | N/O |
| **5** | 15.95 | 2.29 | 0.83 | 0.59 |
| **6** | 16.99 | 2.11 | 0.64 | 0.83 |
| **7** | 12.51 | 1.57 | 0.42 | 0.73 |
| **8** | 8.83 | 1.10 | 0.30 | 0.59 |
| **9** | 4.65 | 0.59 | 0.14 | 0.34 |
| **10** | 2.50 | 0.22 | N/O | 0.16 |
| **11** | 1.04 | N/O | N/O | N/O |

**Figure S51:** GC chromatogram of **3d**.

**Table 2.7-Relative %fractions of compound 3d**

| ***n*** | **1-alkene-R** | **H-alkane** | **Int-alkenes** | **1-alkene-Et** |
| --- | --- | --- | --- | --- |
| **3** | 4.29 | 5.04 | 0.77 | N/O |
| **4** | 7.75 | 0.69 | 0.88 | N/O |
| **5** | 10.83 | 1.13 | 1.32 | 0.68 |
| **6** | 12.97 | 1.47 | 1.20 | 0.77 |
| **7** | 13.88 | 1.40 | 1.18 | 0.95 |
| **8** | 10.61 | 1.32 | 0.42 | 1.01 |
| **9** | 7.89 | 0.75 | 0.34 | 0.86 |
| **10** | 5.38 | N/O | N/O | 0.68 |
| **11** | 2.10 | N/O | N/O | N/O |

**^1^H 2D COSY Spectra**

**Figure S52:** ^1^H COSY NMR spectrum of **3a_1_** (400 MHz, 25 °C, *Chloroform-*d*)

**DSC Traces**

**Figure S53.** DSC trace of **3a_1_** (exotherm up). Heat/cool/heat cycle with heating ramp of 10°C/min and cooling ramp of 5 °C/min from -50 to 250 °C.

**Figure S54.** DSC trace of **3a_2_** (exotherm up). Heat/cool/heat cycle with heating ramp of 10°C/min and cooling ramp of 5 °C/min from -50 to 250 °C.

4

**Figure S55.** DSC trace of **3a_3_** (exotherm up). Heat/cool/heat cycle with heating ramp of 10°C/min and cooling ramp of 5 °C/min from -50 to 250 °C.

**Figure S56.** DSC trace of **3a_4_** (exotherm up). Heat/cool/heat cycle with heating ramp of 10°C/min and cooling ramp of 5 °C/min from -50 to 250 °C.

**Figure S57.** DSC trace of **3b** (exotherm up). Heat/cool/heat cycle with heating ramp of 10°C/min and cooling ramp of 5 °C/min from -50 to 250 °C.

**Figure S58.** DSC trace of **3c** (exotherm up). Heat/cool/heat cycle with heating ramp of 10°C/min and cooling ramp of 5 °C/min from -50 to 250 °C.

**Figure S59.** DSC trace of **3d** (exotherm up). Heat/cool/heat cycle with heating ramp of 10°C/min and cooling ramp of 5 °C/min from -50 to 250 °C.

**Figure S60.** DSC trace of **Run 1** (exotherm up). Heat/cool/heat cycle with heating ramp of 10°C/min and cooling ramp of 5 °C/min from -70 to 250 °C.

**Figure S61.** DSC trace of **Run 2** (exotherm up). Heat/cool/heat cycle with heating ramp of 10°C/min and cooling ramp of 5 °C/min from -70 to 250 °C.

**Figure S62.** DSC trace of **Run 3** (exotherm up). Heat/cool/heat cycle with heating ramp of 10°C/min and cooling ramp of 5 °C/min from -70 to 250 °C.

**Figure S63.** DSC trace of **Run 4** (exotherm up). Heat/cool/heat cycle with heating ramp of 10°C/min and cooling ramp of 5 °C/min from -70 to 250 °C.

**Figure S64.** DSC trace of **Run 5** (exotherm up). Heat/cool/heat cycle with heating ramp of 10°C/min and cooling ramp of 5 °C/min from -50 to 250 °C.

**Figure S65.** DSC trace of **Run 6** (exotherm up). Heat/cool/heat cycle with heating ramp of 10°C/min and cooling ramp of 5 °C/min from -50 to 250 °C.

**Figure S66.** DSC trace of **Run 7** (exotherm up). Heat/cool/heat cycle with heating ramp of 10°C/min and cooling ramp of 5 °C/min from -50 to 250 °C.

**Figure S67.** DSC trace of **Run 8** (exotherm up). Heat/cool/heat cycle with heating ramp of 10°C/min and cooling ramp of 5 °C/min from -50 to 250 °C.

**Figure S68.** DSC trace of **Run 9** (exotherm up). Heat/cool/heat cycle with heating ramp of 10°C/min and cooling ramp of 5 °C/min from -70 to 250 °C.

**Figure S69.** DSC trace of **Run 10** (exotherm up). Heat/cool/heat cycle with heating ramp of 10°C/min and cooling ramp of 5 °C/min from -70 to 250 °C.

**Figure S70.** DSC trace of **Run 11** (exotherm up). Heat/cool/heat cycle with heating ramp of 10°C/min and cooling ramp of 5 °C/min from -50 to 250 °C.

**Figure S71.** DSC trace of **Run 12** (exotherm up). Heat/cool/heat cycle with heating ramp of 10°C/min and cooling ramp of 5 °C/min from -70 to 250 °C.

**Figure S72.** DSC trace of **Run 13** (exotherm up). Heat/cool/heat cycle with heating ramp of 10°C/min and cooling ramp of 5 °C/min from -70 to 250 °C.

**Figure S73.** DSC trace of **Run 14** (exotherm up). Heat/cool/heat cycle with heating ramp of 10°C/min and cooling ramp of 5 °C/min from -70 to 250 °C.

**Figure S74.** DSC trace of **Run 15** (exotherm up). Heat/cool/heat cycle with heating ramp of 10°C/min and cooling ramp of 5 °C/min from -50 to 250 °C.

**TGA Traces**

**Figure S75.** TGA trace of **3a_1_**, sample heated from room temperature at 20°C/min to 800°C.

**Figure S76.** TGA trace of **3a_2_**, sample heated from room temperature at 20°C/min to 800°C.

**Figure S77.** TGA trace of **3a_3_**, sample heated from room temperature at 20°C/min to 800°C.

**Figure S78.** TGA trace of **3a_4_**, sample heated from room temperature at 20°C/min to 800°C.

**Figure S79.** TGA trace of **3b**, sample heated from room temperature at 20°C/min to 800°C.

**Figure S80.** TGA trace of **3c**, sample heated from room temperature at 20°C/min to 800°C.

**Figure S81.** TGA trace of **3d**, sample heated from room temperature at 20°C/min to 800°C.

**Figure S82.** TGA trace of **Run 1**, sample heated from room temperature at 20°C/min to 800°C.

**Figure S83.** TGA trace of **Run 2**, sample heated from room temperature at 20°C/min to 800°C.

**Figure S84.** TGA trace of **Run 3**, sample heated from room temperature at 20°C/min to 800°C.

**Figure S85.** TGA trace of **Run 4**, sample heated from room temperature at 20°C/min to 800°C.

**Figure S86.** TGA trace of **Run 5**, sample heated from room temperature at 20°C/min to 800°C.

**Figure S87.** TGA trace of **Run 6**, sample heated from room temperature at 20°C/min to 800°C.

**Figure S88.** TGA trace of **Run 7**, sample heated from room temperature at 20°C/min to 800°C.

**Figure S89.** TGA trace of **Run 8**, sample heated from room temperature at 20°C/min to 800°C.

**Figure S90.** TGA trace of **Run 9**, sample heated from room temperature at 20°C/min to 800°C.

**Figure S91.** TGA trace of **Run 10**, sample heated from room temperature at 20°C/min to 800°C.

**Figure S92.** TGA trace of **Run 11**, sample heated from room temperature at 20°C/min to 800°C.

**Figure S93.** TGA trace of **Run 12**, sample heated from room temperature at 20°C/min to 800°C.

**Figure S94.** TGA trace of **Run 13**, sample heated from room temperature at 20°C/min to 800°C.

**Figure S95.** TGA trace of **Run 14**, sample heated from room temperature at 20°C/min to 800°C.

**Figure S96.** TGA trace of **Run 15**, sample heated from room temperature at 20°C/min to 800°C.

**SEC Traces**

**Figure S97.** SEC trace of Run **1**.

**Figure S98.** SEC trace of Run **2**.

**Figure S99.** SEC trace of Run **3**.

**Figure S100.** SEC trace of Run **4**.

**Figure S101.** SEC trace of Run **5**.

**Figure S102.** High Temperature SEC trace of Run **6**.

**Figure S103.** High Temperature SEC trace of Run **7**.

**Figure S104.** High Temperature SEC trace of Run **8**.

**Figure S105.** SEC trace of Run **9.**

**Figure S106.** SEC trace of Run **10.**

**Figure S107.** High Temperature SEC trace of Run **11**.

**Figure S108.** High Temperature SEC trace of Run **12**.

**Figure S109.** High Temperature SEC trace of Run **13**.

**Figure S110.** High Temperature SEC trace of Run **14**.

**Figure S111.** High Temperature SEC trace of Run **15**.

**References**

[1] B. R. S. Kuzminski, W. R. Burgenson, L. R. Sita, *ACS Catal.* **2025**, *15,* 12897–12904.

[2] W. Zhang, L. R. Sita, *J. Am. Chem. Soc*. **2008**, 130, 442-443.

[3] K. C. Jayaratne, L. R. Sita, *J. Am. Chem. Soc.* **2000**, *122*, 958-959.

[4] M. A. Wallace, P. Y. Zavalij, L. R. Sita, *ACS Catal*. **2020**, 10, 8496–8502.

[5] G. W. Coates, R. M. Waymouth, *J. Am. Chem. Soc*. **1991**, *113*, 6270-6271.
